# Syphilitic Uveitis-Associated Chiasmal Optic Neuritis Presenting As Bitemporal Hemianopia-Like Visual Field Defects

**DOI:** 10.7759/cureus.100656

**Published:** 2026-01-03

**Authors:** Tomoya Ishibe, Mami Otsuka, Maho Itotani, Atsunobu Takeda

**Affiliations:** 1 Department of Ophthalmology, Oita University, Oita, JPN

**Keywords:** acute syphilitic posterior placoid chorioretinitis, bitemporal hemianopia, chiasmal optic neuritis, oral amoxicillin hydrate therapy, syphilitic uveitis

## Abstract

Syphilis is a systemic infectious disease that can involve both ocular tissues and the central nervous system, with syphilitic uveitis representing one of its ocular manifestations. We report a rare case of syphilitic uveitis occurring in conjunction with chiasmal optic neuritis. A 48-year-old man presented with blurred vision in the right eye and was found to have bilateral acute syphilitic posterior placoid chorioretinitis with vitreous opacity in the left eye. Visual field testing demonstrated bitemporal hemianopia-like defects, and magnetic resonance imaging revealed enhancement of the optic chiasm. Serologic testing confirmed syphilis. Oral amoxicillin therapy resulted in complete resolution of the inflammation and recovery of vision. This case highlights that bitemporal hemianopia in patients with syphilitic uveitis may reflect concurrent chiasmal involvement rather than uveitic pathology alone, underscoring the importance of early diagnosis and appropriate treatment are crucial for achieving favorable visual outcomes.

## Introduction

Syphilis is a sexually transmitted systemic disease caused by *Treponema pallidum*, which penetrates minute abrasions of the skin or mucous membranes and disseminates hematogenously via the lymphatic system [1]. In Japan and developed countries, including the United States, the number of patients with syphilis has been increasing in recent years. By 2022, this number had reached approximately 13,000 cases [2]. Syphilitic uveitis can occur at any stage of syphilis and presents with a wide variety of ocular manifestations, including blepharoconjunctivitis, interstitial keratitis, iridocyclitis, vitreous opacities, retinal vasculitis, neuroretinitis, and acute syphilitic posterior placoid chorioretinitis (ASPPC); thus, making a diagnosis is often challenging [3]. Optic nerve involvement occurs in 78% of patients with syphilitic uveitis [4]. In patients with ASPPC, visual field defects are typically reported as central scotomas [5,6]. In contrast, bitemporal hemianopia suggests involvement of the optic chiasm rather than primary ocular pathology. Reports of syphilitic chiasmal optic neuritis (ON) remain limited and are largely confined to isolated clinicopathological or radiological observations [7,8]. Herein, we report a rare case of syphilitic uveitis co-associated with chiasmal ON, presenting with bitemporal hemianopia-like visual field defects. This case highlights the importance of recognizing that visual field abnormalities in patients with ocular syphilis may reflect concurrent intracranial involvement rather than uveitic changes alone, thereby warranting prompt neuroimaging.

## Case presentation

A 48-year-old man presented with blurred vision in the right eye. His symptoms had begun 10 days before visiting a local ophthalmologist, who then referred him to the Department of Ophthalmology at Oita University Hospital, Yufu, Japan, 13 days after symptom onset. No fever, rash, or lymphadenopathy was observed, and no endocrine or other neurologic symptoms were identified.

His best-corrected visual acuity (BCVA) was 0.8 oculus dexter (OD) and 1.2 oculus sinister. The intraocular pressure was 10 mmHg bilaterally. The pupillary light reflexes were brisk; however, a relative afferent pupillary defect (RAPD) was present in the right eye. No inflammatory findings were observed in the anterior segment or media of the right eye; however, mild vitreous opacity was observed in the left eye.

**Figure 1:**
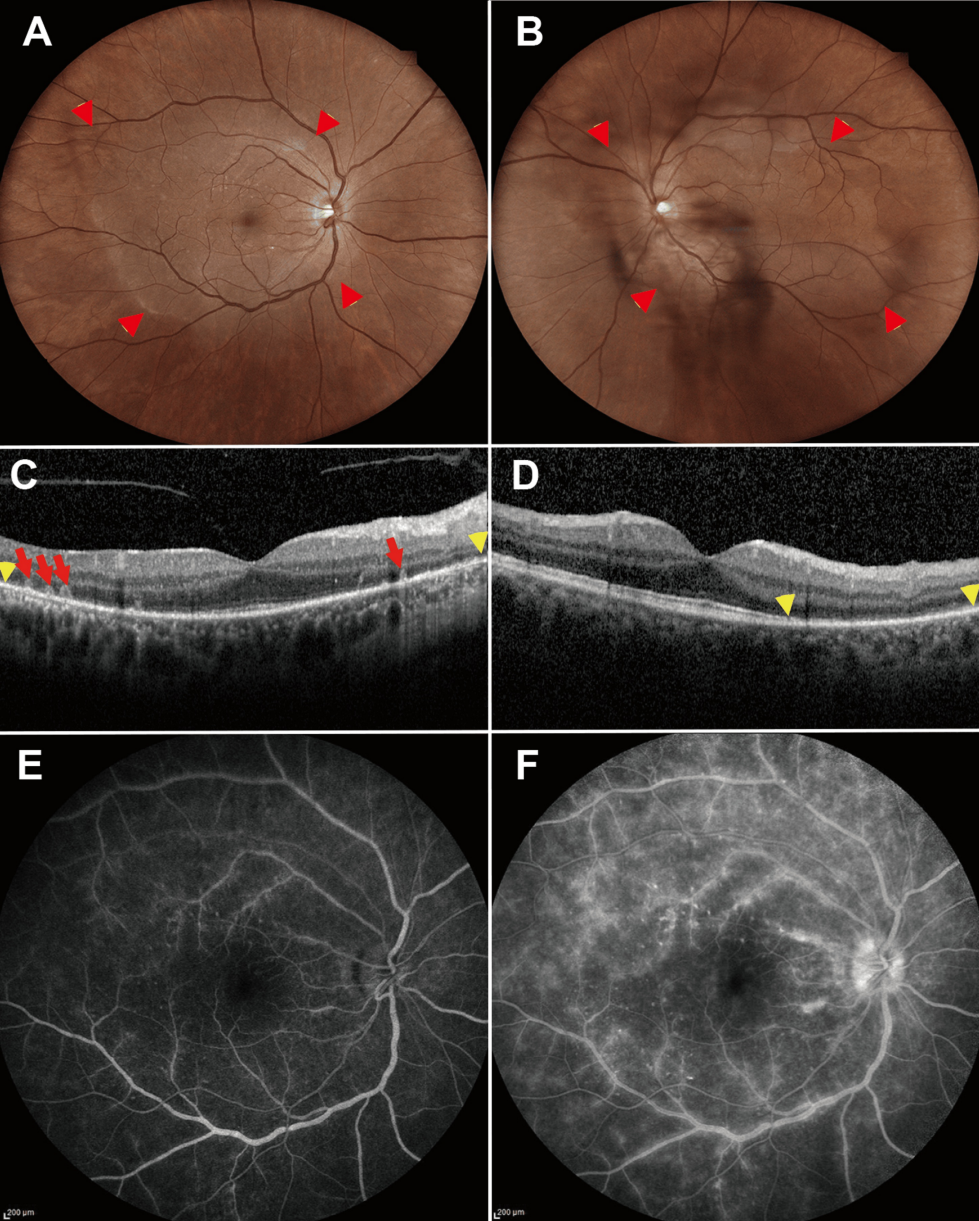
Representative multimodal images of acute syphilitic posterior placoid chorioretinitis in the patient (A) Left eye and (B) right eye. Color fundus photographs show yellow-white placoid lesions at the posterior pole (red arrowheads). Optical coherence tomography images reveal hyperreflective foci within the photoreceptor layer in the right eye (C, red arrows) and disruption of the ellipsoid zone in both eyes (C and D, between the yellow arrowheads). Fluorescein angiography of the right eye shows (E) early-phase hyperfluorescence of the posterior pole vessels and (F) late-phase fern-like leakage corresponding to the sites of the lesions.

The fundus examination revealed a disc-shaped yellow-white lesion, measuring approximately six disc diameters, centered on the macula of both eyes (Figures 1A-1B). Optical coherence tomography (OCT) showed loss of the ellipsoid zone (EZ) at the macula of both eyes, vertical hyperreflective columns extending from the retinal pigment epithelium (RPE) into the outer retina from in the temporal macula of the right eye (Figure 1C), and loss of the EZ at the temporal lesions of the macula in the left eye (Figure 1D). Fluorescein angiography revealed a fern-like hyperfluorescent leakage at the posterior pole of both eyes in the early phase (Figure 1E), with late-phase leakage enhancement and optic disc hyperfluorescence (Figure 1F). The critical flicker fusion frequency was 37 Hz in both eyes.

Chest radiographic findings were unremarkable and showed no findings suggestive of sarcoidosis. C-reactive protein levels were mildly elevated (Table 1). Serum angiotensin-converting enzyme (ACE) and lysozyme levels were within the normal range (Table 1).

**Table 1:** Results of blood tests at the initial visit CRP: C-reactive protein; TP: total protein; T-Bil: total bilirubin; AST: aspartate aminotransferase; ALT: alanine aminotransferase; ALP: alkaline phosphatase; γ-GTP: gamma-glutamyl transpeptidase; CK: creatine kinase; AMY: amylase; BUN: blood urea nitrogen; Cre: creatinine; UA: uric acid; Na: sodium; K: potassium; Cl: chloride; Ca: calcium; Glu: glucose; ACE: angiotensin-converting enzyme; WBC: white blood cell count; RBC: red blood cell count; Hb: hemoglobin; Plt: platelet count; ESR: erythrocyte sedimentation rate; sIL-2R: soluble interleukin-2 receptor; TSH: thyroid-stimulating hormone; FT3: free triiodothyronine; anti-CCP antibody: anti-cyclic citrullinated peptide antibody; anti-dsDNA antibody: anti-double-stranded DNA antibody; SS-A antibody: anti-Sjögren’s syndrome-related antigen A (Ro) antibody; MPO-ANCA: myeloperoxidase antineutrophil cytoplasmic antibody; PR3-ANCA: proteinase-3 antineutrophil cytoplasmic antibody; HBs antigen: hepatitis B surface antigen; HCV: hepatitis C virus; RPR: rapid plasma reagin; TPHA: *Treponema pallidum* hemagglutination assay; HTLV-1 antibody: human T-cell leukemia virus type 1 antibody; HIV: human immunodeficiency virus; HSV-IgM: herpes simplex virus immunoglobulin M; VZV-IgM: varicella-zoster virus immunoglobulin M; CMV-IgM: cytomegalovirus immunoglobulin M

Items	Patient value	Reference/Normal range
CRP	0.36 mg/dL	<0.3 mg/dL
TP	8.1 g/dL	6.6-8.1 g/dL
T-Bil	1.8 mg/dL	0.2-1.2 mg/dL
AST	25 IU/L	13-30 IU/L
ALT	35 IU/L	10-40 IU/L
ALP	107 IU/L	38-113 IU/L
γ-GTP	89 IU/L	10-50 IU/L
CK	135 IU/L	62-287 IU/L
AMY	68 IU/L	37-125 IU/L
BUN	13 mg/dL	8-20 mg/dL
Cre	1.1 mg/dL	0.6-1.2 mg/dL
UA	7.7 mg/dL	3.0-7.0 mg/dL
Na	140 mEq/L	135-145 mEq/L
K	4.1 mEq/L	3.5-5.0 mEq/L
Cl	106 mEq/L	98-107 mEq/L
Ca	9.3 mg/dL	8.5-10.5 mg/dL
Glu	103 mg/dL	70-109 mg/dL
ACE	12.4 U/L	8.3–21.4 U/L
Lysozyme	10.0 μg/mL	5.0-10.2 μg/mL
WBC	6610 /μL	4000-10000 /μL
RBC	5.08×10^6^/μL	4.5-5.9×10^6^/μL
Hb	15 g/dL	13.5-17.5 g/dL
Plt	30×10^4^/μL	15-40×10^4^/μL
ESR	48 mm/h	<15 mm/h
sIL-2R	524 U/mL	122-496 U/mL
TSH	3.15 μIU/mL	0.4-4.0 μIU/mL
FT3	3.32 pg/mL	2.0-4.4 pg/mL
Anti-CCP antibody	<0.6 U/mL	<4.5 U/mL
Anti-dsDNA antibody	3.5 IU/mL	<30 IU/mL
SS-A antibody	<1.0 U/mL	<10 U/mL
MPO-ANCA	1.0 U/mL	<3.5 U/mL
PR3-ANCA	1.9 U/mL	<3.5 U/mL
Rheumatoid factor	<5 IU/mL	<15 IU/mL
HBs antigen	Negative	Negative
HCV antibody	Negative	Negative
RPR	1:128	Negative
TPHA	Positive	Negative
HTLV-1 antibody	1.0	<1.0
HIV antibody	0.15	Negative
HSV-IgM	Negative	Negative
VZV-IgM	Negative	Negative
CMV-IgM	Negative	Negative

Serological tests revealed an elevated rapid plasma reagin (RPR) titer and positive results for *T. pallidum* antibodies (Table 1). The patient tested negative for antibodies against human immunodeficiency virus (Table 1). Cerebrospinal fluid (CSF) analysis revealed negative RPR and positive *T. pallidum* assay (Table 2).

**Table 2:** Results of cerebrospinal fluids at the initial visit Bold values indicate abnormal values. RPR: rapid plasma reagin; TPHA: *Treponema pallidum* hemagglutination assay

Parameter	Patient value	Reference range
Specific gravity	1.007	1.006-1.008
Cell Count	12/μL	0-5/μL
Polymorphonuclear cells	3%	<10%
Mononuclear cells	97%	>90%
Protein	63 mg/dL	15-45 mg/dL
Glucose	59 mg/dL	50-80 mg/dL
RPR	Negative	Negative
TPHA	Positive	Negative

**Figure 2:**
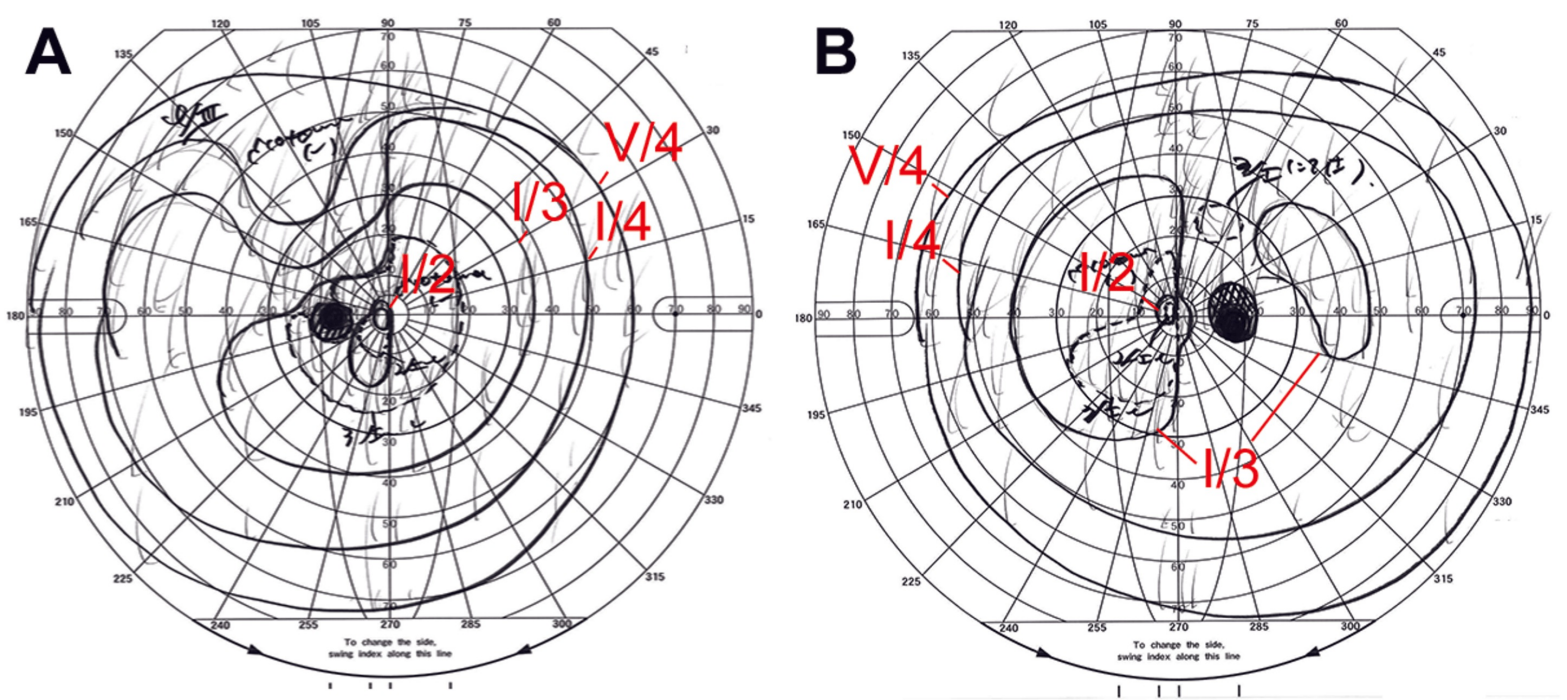
Goldmann visual field test results at initial visit (A) Left eye and (B) right eye. Goldmann perimetry demonstrates bitemporal hemianopia-like with enlarged blind spots, which is consistent with chiasmal optic neuritis. The I/2, I/3, I/4, and V/4 isopters are labeled red for clarity.

**Figure 3:**
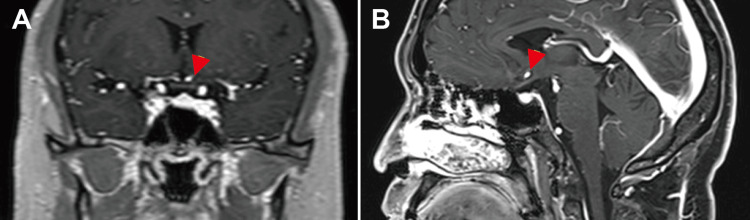
Fat-suppressed T1-weighted post-contrast-enhanced magnetic resonance imaging of the brain (A) The coronal and (B) sagittal fat-suppressed T1-weighted post-contrast magnetic resonance images demonstrate an enhancement at the optic chiasm (red arrowheads), indicating inflammation involving the chiasmal region and consistent with active neuritis.

Goldmann perimetry revealed a bitemporal hemianopia-like defect and enlarged blind spots in both eyes (Figures 2A-2B). Contrast-enhanced magnetic resonance imaging (MRI) revealed enhancement of the optic chiasm (Figures 3A-3B). The patient was diagnosed with syphilitic uveitis, including ASPPC and chiasmal ON.

**Figure 4:**
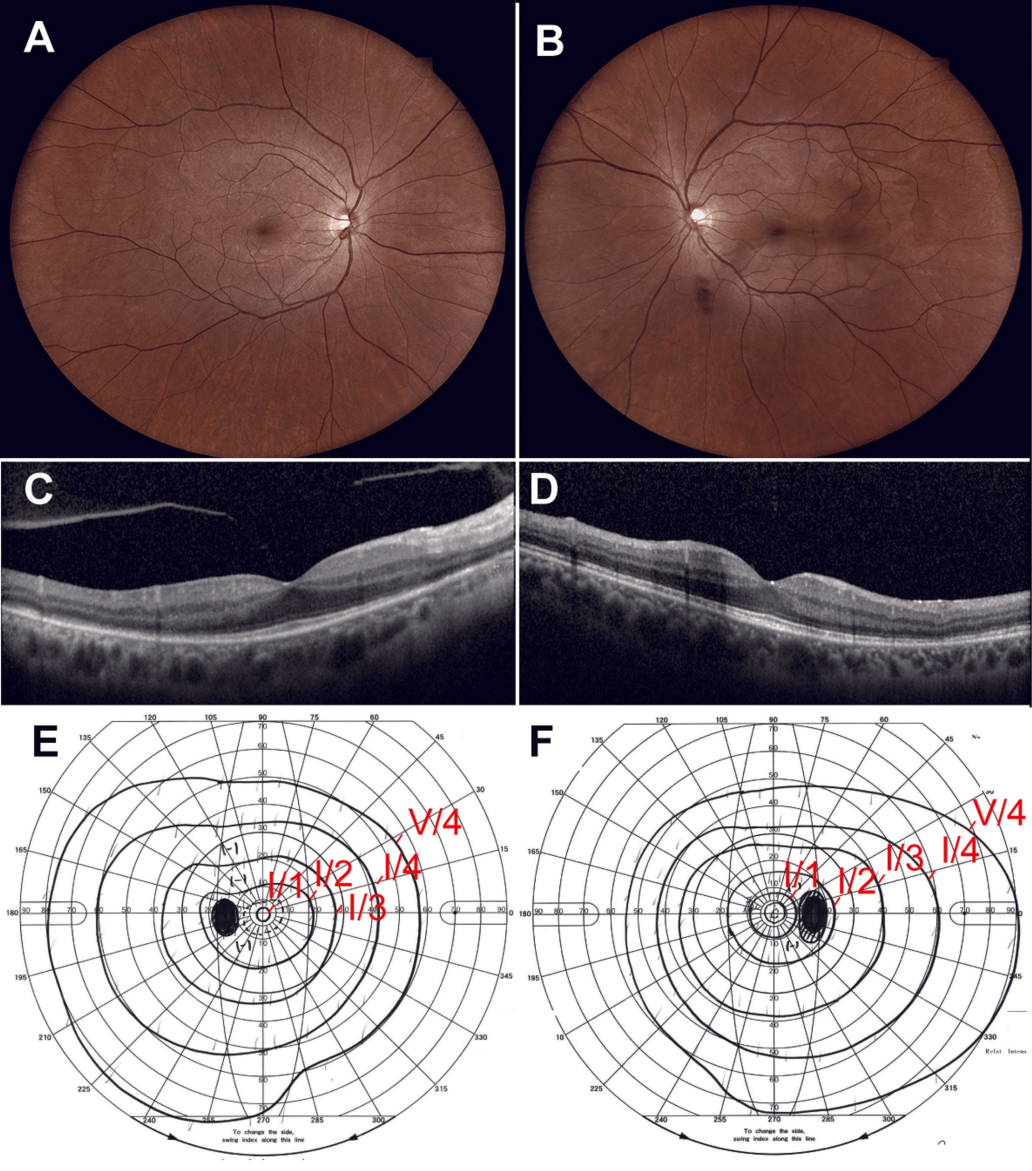
Optical coherence tomography, fundus images, and Goldman perimetry three months after antibiotic treatment (A, B) Color fundus photographs of the left and right eye, respectively, show the disappearance of yellow-white placoid lesions at the posterior pole. (C, D) Corresponding optical coherence tomography scans show restoration of the ellipsoid zone after treatment. (E, F) Goldmann visual field examinations of the left and right eyes, respectively, demonstrate improvement in visual field sensitivity using the I/1, I/2, I/3, I/4, and V/4 isopters, which are labeled red for clarity.

Oral amoxicillin hydrate was initiated 20 days after symptom onset at a dosage of 1,500 mg/day. Two weeks later, the RAPD in the right eye had resolved, and the vitreous opacity in the left eye had improved. After one month, his BCVA improved to 1.2 OD, and the disc-shaped yellow-white lesions at the posterior pole had resolved (Figure 4A). OCT revealed resolution of outer retinal irregularities and RPE elevation (Figure 4B). The visual field defects also improved. After three months of treatment, oral amoxicillin hydrate therapy was discontinued because the serum RPR titer had decreased to 1:32. No recurrence was observed.

## Discussion

Visual field loss is often the first sign of optic chiasm syndrome, most commonly presenting as unilateral or bitemporal hemianopia from chiasmal tumors, such as pituitary adenoma, although it may rarely occur with chiasmal ON [9]. Chiasmal ON has been described in systemic lupus erythematosus, aquaporin-4 (AQP4) antibody-related diseases, and infections caused by the Epstein-Barr and varicella-zoster viruses [10]. Chiasmal ON is more common in patients with AQP4 antibody-associated ON than in patients with demyelinating ON diagnosed with contrast-enhanced MRI, highlighting the usefulness of MRI in cases of bitemporal hemianopia without tumor [11]. In our patient, the visual field defect was a bitemporal hemianopia-like pattern; therefore, intracranial disease was suspected. Chiasmal ON was subsequently confirmed with fat-suppressed T1-weighted post-contrast MRI. These results suggested that neuroimaging should be examined when bitemporal hemianopia-like visual field defects are detected in patients with syphilitic uveitis.

ASPPC is a subtype of posterior uveitis associated with syphilis, characterized by a disc-shaped yellow-white lesion at the macula, disruption of the inner segment/outer segment junction, and hyperreflective thickening of RPE on OCT [1,12]. The underlying pathology of ASPPC is presumed to involve localized inflammation triggered by *T. pallidum* infection, leading to the degeneration and loss of photoreceptor outer segments, which are visualized on OCT as a disruption of or the disappearance of the EZ [13]. Visual field defects in patients with ASPPC have been reported to manifest as central scotomas [5,6]. However, this patient did not exhibit a central scotoma at the initial visit, further supporting the atypical nature of the visual field findings.

In patients with syphilitic uveitis, initiating treatment more than 12 weeks after symptom onset is associated with poor visual outcomes [14]. In the present case, the reduction in visual acuity in the right eye was mild (0.8) at presentation. The visual field defect improved after treatment (Figures 4E-4F), suggesting that therapy was initiated sufficiently early to prevent photoreceptor damage and the subsequent development of a detectable central visual field defect.

Syphilitic uveitis can occur at any stage of the disease. No universally standardized treatment guidelines exist for syphilis. The Centers for Disease Control and Prevention (CDC) recommends that anterior uveitis in early syphilis is treated with a single intramuscular injection of 2.4 million units of benzathine penicillin G, whereas posterior uveitis, panuveitis, and optic nerve involvement should be managed as neurosyphilis, for which intravenous aqueous crystalline penicillin G is the standard care [15]. In Japan, the 2020 Sexually Transmitted Diseases Diagnostic and Treatment Guidelines from the Japanese Society of Sexually Transmitted Diseases [16], revised in 2023, recommend using oral amoxicillin or intramuscular benzathine penicillin G as the first-line therapies for syphilis [16]. Bazewicz et al. reported that more than one-half of patients met the CDC 2015 criteria for neurosyphilis, approximately 70% of the patients had CSF abnormalities, and all had high serum RPR titers [17]. In the present case, systemic symptoms were absent, and the CSF RPR was negative, suggesting a low likelihood of neurosyphilis. Therefore, we selected oral amoxicillin for the treatment. Neither neurosyphilis nor the recurrence of ocular inflammation was observed during follow-up.

This study has some limitations. The findings from a single case cannot be generalized to all patients with syphilis or chiasmal ON. Although the CSF RPR test result was negative, the possibility of a false-negative result due to the prozone phenomenon cannot be completely excluded. Additionally, a follow-up MRI was not performed; therefore, radiologic resolution of the chiasmal lesion could be evaluated. Moreover, the efficacy of oral amoxicillin, compared with the standard intravenous penicillin regimen, remains uncertain, and long-term outcomes have not been evaluated. Nevertheless, this case highlights the importance of an early diagnosis and prompt treatment of ocular syphilis with possible intracranial involvement.

## Conclusions

This case illustrates a rare manifestation of syphilitic uveitis complicated by chiasmal ON, presenting with bitemporal hemianopia-like visual field defects. The findings underscore that ocular syphilis can involve intracranial structures even in the absence of systemic symptoms or CSF abnormalities. Recognition of visual field patterns that are inconsistent with fundus findings should prompt neuroimaging to exclude central nervous system involvement. In this patient, early diagnosis and timely antibiotic therapy are essential to prevent irreversible visual loss and achieve a favorable functional outcome. However, the therapeutic approach described herein reflects an individualized treatment decision based on national guidelines and the specific clinical context; therefore, it should not be interpreted as evidence that oral amoxicillin is equivalent to standard neurosyphilis regimens. Given the single-case nature of this report and the uncertainty regarding long-term outcomes, caution is warranted in generalizing treatment strategies. Continued awareness of the variable and sometimes deceptive presentations of ocular syphilis remains critical for clinicians in ophthalmology and neurology.
